# Comparative Study of Several Machine Learning Algorithms for Classification of Unifloral Honeys

**DOI:** 10.3390/foods10071543

**Published:** 2021-07-03

**Authors:** Fernando Mateo, Andrea Tarazona, Eva María Mateo

**Affiliations:** 1 Department of Electronic Engineering, ETSE, University of Valencia, 46100 Burjasot, Spain; 2 Department of Microbiology and Ecology, University of Valencia, 46100 Burjasot, Spain; Andrea.tarazona@uv.es; 3 Department of Microbiology, School of Medicine, University of Valencia, 46010 Valencia, Spain; Eva.mateo@uv.es

**Keywords:** machine learning, unifloral honeys, botanical origin, physicochemical parameters, classification

## Abstract

Unifloral honeys are highly demanded by honey consumers, especially in Europe. To ensure that a honey belongs to a very appreciated botanical class, the classical methodology is palynological analysis to identify and count pollen grains. Highly trained personnel are needed to perform this task, which complicates the characterization of honey botanical origins. Organoleptic assessment of honey by expert personnel helps to confirm such classification. In this study, the ability of different machine learning (ML) algorithms to correctly classify seven types of Spanish honeys of single botanical origins (rosemary, citrus, lavender, sunflower, eucalyptus, heather and forest honeydew) was investigated comparatively. The botanical origin of the samples was ascertained by pollen analysis complemented with organoleptic assessment. Physicochemical parameters such as electrical conductivity, pH, water content, carbohydrates and color of unifloral honeys were used to build the dataset. The following ML algorithms were tested: penalized discriminant analysis (PDA), shrinkage discriminant analysis (SDA), high-dimensional discriminant analysis (HDDA), nearest shrunken centroids (PAM), partial least squares (PLS), C5.0 tree, extremely randomized trees (ET), weighted k-nearest neighbors (KKNN), artificial neural networks (ANN), random forest (RF), support vector machine (SVM) with linear and radial kernels and extreme gradient boosting trees (XGBoost). The ML models were optimized by repeated 10-fold cross-validation primarily on the basis of log loss or accuracy metrics, and their performance was compared on a test set in order to select the best predicting model. Built models using PDA produced the best results in terms of overall accuracy on the test set. ANN, ET, RF and XGBoost models also provided good results, while SVM proved to be the worst.

## 1. Introduction

Honey is a natural food appreciated worldwide with high nutritional value that provides many health benefits [1,2]. Honey is defined by the European Union (EU) as “the natural sweet substance produced by *Apis mellifera* bees from the nectar of plants or from secretions of living parts of plants or excretions of plant-sucking insects or the living parts of plants, which the bees collect, transform by combining with specific substances of their own, deposit, dehydrate, store, and leave in honey combs to ripen and mature” [3]. The EU regulations concerning honey are included mainly in the 2001/110/EC Council Directive [4], further amended by the 2014/63/EU Directive [5]. The composition criteria for honey placed on the market or used in any product intended for human consumption are stated in Annex II of [4]. The Codex standard for honey adopted by the Codex Alimentarius Commission in 1981 was revised in 1987 and 2001, has served as a basis for national legislations in some countries and has voluntary application [6]. Honey is a very rich food product that contains water, sugars (mainly fructose and glucose, but also di- and trisaccharides), hydroxymethyl furfural and other compounds at low levels such as minerals, amino acids, proteins (including enzymes), aromatic acids, esters, aroma components and flavonoids [1,2]. Naturally, bees forage the flowers they can access. Hence, the honey produced mostly has a blend of flavors and is commonly sold in the market simply as honey or mixed-flower honey. However, when the nectar is taken predominantly from a single type of flower, the honey produced has characteristic organoleptic properties, adding to its commercial value. Many consumers appreciate these particular sensorial properties very much, which increase these honeys’ price with respect to other types of honey. Moreover, honeydew honeys are especially appreciated by consumers in Central Europe (Germany, Switzerland and Austria). Denominations of botanical origin are extensively used on the honey market as they offer consumers the choice among a variety of different typical products, paying prices depending on local consumer preferences [7]. The existing international norms and regulations do not specify the characteristics of unifloral honeys, although limits for moisture content, sugar content or electrical conductivity are different for honeys originated from some botanical origins [2]. Authentication of food products is of great concern in the context of food safety and quality. In recent years, interest in honey authenticity in relation to botanical or geographical origin and adulteration has increased. Due to the huge variety of different floral sources normally attainable by bees for foraging and to the great diversity within plant species, which is influenced by the climatic and growing conditions, the parameters used for characterizing unifloral honeys do not exhibit typical values but are defined in rather large, often overlapping ranges [7]. The differences observed in honey composition depend on a variety of factors, such as the region, season, nectar source, beekeeping practices and harvest period [8].

Classically, the determination of the botanical origin of honeys has been performed by melissopalynological methods. The fundamentals of this methodology were established many years ago [9,10], but it has been used for years. Usually, honey is considered mainly from one plant if the pollen frequency of that plant is >45%. Pollen grains from anemophilous plants and plants with nectarless flowers are excluded in the calculation of the percentages. Moreover, pollen grains from some species are under- or over-represented in relation to the nectar their flowers yield. For unifloral honeys with under-represented pollen, the minimum percentage of the taxon that gives the honey its name ranges 10–30%; for those with over-represented pollen, the minimum percentage can be 80–90%. This technique is useless in the case of honey filtration. Notwithstanding, interpretation of pollen analysis data may be difficult in some cases, and the counting and identification of pollen grains depend greatly on the skill and performance of the analyst [11]. Sensory properties (color, aroma, flavor) can help to ascribe a honey sample to a given botanical origin, but, due to subjectivity, well-trained personnel are needed. However, the sensory properties of a honey can vary with time and thermal treatment while maintaining the floral origin. Organoleptic properties have been considered, together with pollen analysis, key to performing the classification of unifloral honeys. Methods based on physicochemical properties of honey have been developed for the accurate classification of these honeys with the help of suitable statistical treatments [11]. Generally, no single parameter has proved useful to characterize the botanical origin of honey, except the methyl anthranilate content, which is characteristic of citrus honey [12,13]. Assayed parameters have been honey color (measured using CIE-1931 xyL or CIE-1976 LAB chromatic coordinates) [14], the carbohydrate profile [15,16,17], volatile organic compounds [16,18,19,20,21,22] or the amino acid profile [18,23].

Even when differences among honeys from distinct botanical sources are found using only a profile of a single class of compounds (sugars, amino acids, volatiles, etc.) or characteristics, a thorough characterization of the botanical origin of honeys is not achieved. Thus, sets of different parameters, either physicochemical or sensorial, or both, sometimes with the pollen spectrum and usually involving statistical (chemometric) techniques, such as cluster analysis, principal component analysis (PCA) and linear discriminant analysis (LDA), have been considered. Parameters tested together with this aim have been water content, pH, acidity, electrical conductivity, some carbohydrates, color, volatile compounds, amino acids, phenolic compounds, mineral elements, etc. [11,22,23,24,25,26]. Even when the chemical composition of honey is associated with its botanical and geographical origin, some processes, such as heating, storing or the extraction techniques, can alter the initial volatile composition [22], which affects the volatile fingerprint of unifloral honeys and hence organoleptic properties. Other classification approaches lie in the use of nondestructive techniques applied to honey samples. In this way, attenuated total reflectance Fourier-transform infrared spectroscopy (ATR-FTIR) of unifloral honeys is a technique that, after treatment by PCA and further treatment of the principal components by means of a machine learning (ML) algorithm such as support vector machine (SVM), proved useful for the characterization of honey origin [27]. The potential application of other spectroscopy techniques such as visible–near-infrared (VIS–NIR) hyperspectral imaging for the detection of honey flower origin using ML techniques has been reported [28]. PCA was used for dimensionality reduction before ML treatment using three ML algorithms, namely, radial basis function (RBF) network, SVM and random forest (RF), to predict honey floral origin. Furthermore, FT-Raman spectroscopy has shown to be a simple, rapid and nondestructive technique that, in combination with proper PCA or LDA models, could be successfully adopted to identify the botanical origins of some honey types [29,30,31]. The same technique resulted in being useful to detect adulterations of pure beeswax with paraffin or microcrystalline waxes [32]. Nuclear magnetic resonance (NMR) was used for the estimation of the botanical origin of honeys. Due to the complex nature of NMR data, multivariate analysis has been applied to extract the useful information [33]. The application of an electronic nose (E-nose) to parametrize the odor compounds in the form of numeric resistance and further treatment by k-nearest neighbor (k-NN) has been reported [34]. A commercial electronic tongue including seven potentiometric sensors has been applied for the classification of honeys. Botanical classification was performed by PCA, canonical correlation analysis (CCA) and artificial neural network (ANN) modeling on samples of acacia, chestnut and honeydew honeys [35]. The ML algorithms applied to the authentication of the botanical origin of honeys are ANN [35,36], classification and regression trees (CART) [37], k-NN, SVM or RF [27,28,34]. However, the usage of ML techniques is not popular in honey research, and mixed approaches including classical statistics together with ML have been applied in some studies, as indicated in a recent review [38].

The aim of the present study was to carry out a comparative analysis of the application of some ML algorithms to find the most useful to accurately classify rosemary, citrus, lavender, sunflower, heather, eucalyptus and forest unifloral honeys harvested in Spain on the basis of some physicochemical properties (pH, moisture, electrical conductivity, sugars) and color. The classifier algorithms used for this goal were penalized discriminant analysis (PDA), high-dimensional discriminant analysis (HDDA), shrinkage discriminant analysis (SDA), nearest shrunken centroids (PAM), partial least squares (PLS) or decision trees (5.0 tree), extremely randomized trees or Extra Trees (ET), k-NN, SVM, RF and extreme gradient boosted tree (XGBoost).

## 2. Materials and Methods

### 2.1. Honey Samples

The analyzed Spanish honey samples were obtained from beekeepers and traders before processing. They belonged to seven unifloral origins, most of which are very appreciated by consumers worldwide or in countries of Central Europe. They were rosemary (*Rosmarinus officinalis* L.), orange blossom (*Citrus* spp.), lavender (*Lavandula latifolia*, *L. angustifolia*, *L. vera*), sunflower (*Helianthus annuus* L.), heather/bell heather (Ericaceae, mainly *Erica* spp. and *Calluna vulgaris*), eucalyptus (*Eucalyptus globulus* and *E. camaldulensis*) and forest honeys. Honeys were harvested in different regions of Spain, excluding the Balearic and Canary Islands. The approximated coordinates of the areas related to honey harvest are longitude 3°19′ E–7° W and latitude 36° N–43° N. Orange blossom honey was harvested mainly in eastern Spain (Valencian Community) and some provinces of Andalucia. Rosemary and lavender honeys were harvested mainly in central and southeastern Spain (Castilla-la Mancha, Castilla-Leon) during spring (rosemary) and summer (lavender). Heather and bell heather (in the following, heather) honey was harvested in many Spanish regions during March or September. Forest honey was mainly honeydew honey from holm oak (*Quercus ilex* L., *Q. rotundifolia* Lam., *Q. bellota* Desf.) grown in western Spain (Extremadura and Salamanca), and it was harvested during August/September or later. Eucalyptus honey was harvested mainly in provinces located in western Spain (Huelva, Extremadura) and northwestern Spain during September–October. Sunflower honey was collected during summer in southern/central Spain (Andalucía and Castilla-La Mancha). Samples were collected in different years from 2010 to 2014.

Samples were screened by microscopic and sensory analysis (color, aroma, taste) assessment as soon as they arrived at the laboratory. When analysis had to be delayed for more than four weeks, they were stored at −20 °C; otherwise, they were stored at 4–6 °C in the dark.

The samples were assessed microscopically for pollen and honeydew elements (HDE), which are mainly unicellular algae, fungal spores and hyphae. HDE/pollen (from nectariferous plants) ratios higher than three are required for honeydew honeys according to Louveaux et al. [10]. However, an HDE/pollen ratio of 1.5 ± 1.2 (0.3–4) was reported in 167 honeydew honeys from different places in Europe [13]. Studies performed in Spain have found rather low values for such index in oak honeydew honey [39]. Melissopalynology seems not to be useful for classification of Spanish oak honeydew honeys [40]. Other required parameters associated with honeydew honeys are electrical conductivity values >800 µs/cm [4,6] and pH values >4.3 [13], besides acceptable sensory assessment (dark amber color, characteristic taste, lack of crystallization tendency). It is known that in rosemary, lavender and citrus honeys, pollen from the flowers of these plants is not dominant. After screening, some samples were rejected as unifloral. The number of honey samples collected before the initial screening and the number of samples eventually selected for all physicochemical analyses and statistical treatments are indicated in the following relation, where the selected samples are between parenthesis: 27(13) from rosemary, 31(13) from heather, 35(16) from orange blossom, 33(16) from forest, 19(14) from lavender, 23(14) from eucalyptus and 33(14) from sunflower.

### 2.2. Microscopical Analysis

Microscopical analysis of honey sediment was achieved according to the methods of melissopalinology [10] and the Spanish official methods of analysis for honey [41]. Slides were prepared without acetolysis. Briefly, graduated conical centrifuge tubes containing 10× *g* of homogenized honey solved in 20 mL of dilute sulfuric acid were centrifuged for 10 min at 2500 rpm. The supernatant was discarded, and the sediment was washed twice with 10 mL of distilled water and centrifuged. After discarding the supernatant, the sediment was homogenized, and an aliquot was placed on a glass slide, sprouted over an area of 4 cm^2^, dried at 40 °C and mounted with stained glycerin-gelatin. Pollen grains were identified by light microscopy with the aid of non-acetolyzed pollen collection and microphotographs from specialized studies. Usually, 350–500 grains were counted, and they were classified in the following frequency classes: dominant pollen (>45% of the pollen grains counted); secondary pollen (16–45%); important minor pollen (3–15%); minor pollen (1–3%); and present (<1%). For forest honey, HDE were counted apart from pollen grains.

### 2.3. Electrical Conductivity

Electrical conductivity was measured at 20.0 ± 0.1 °C in a 20% (*w*/*v*) solution of honey (dry matter basis) in deionized water with electrical conductivity of <1 µs/cm [41] using a Crison model 525 conductimeter (Crison Instruments, Barcelona, Spain). The cell was previously calibrated at 20.0 °C with a 0.01 M KCl solution. Measurements were carried out in quintuplicate.

### 2.4. Water Content

Water content was determined at 20.0 °C by refractometry according to [41]; this method matches the AOAC 969.38B method [42] cited in [6]. A Bellingham and Stanley standard model Abbe-type refractometer previously calibrated and connected to a thermostatic bath was used. The Chataway tables revised by Wedmore were used to convert refraction indices to percentage of water [41]. Measurements were carried out in triplicate.

### 2.5. pH Measurement

Measurements of pH were performed at 20.0 ± 0.1 °C in a 10% (*w*/*v*) solution of honey in freshly boiled distilled water using a Crison micropH 2000 pH-meter (Crison Instruments, Barcelona, Spain). The pH-meter was calibrated with buffers of pH 6.50 and 3.00 just before measurements, which were conducted in triplicate.

### 2.6. Color

Honeys were liquefied if needed by heating at 50–60 °C, in the case of crystallized samples, and then left to cool at room temperature. Color of liquid honeys was determined by measurement of transmittances at 30 selected wavelengths, on a Shimadzu UV-vis 240 spectrophotometer fitted (Shimadzu Co., Tokyo, Japan). The x, y and L chromatic coordinates from the CIE-1931 (xyL) color system [43] were calculated from the tristimulus values [14]. Transmittance measurements were conducted in triplicate.

### 2.7. Sugars

Sugars were determined by gas chromatographic (GC) separation of the trimethylsilyl (TMS) derivatives (TMS oximes and TMS ethers in the case of non-reducing sugars) in an OV-17 packed column on a Perkin-Elmer Sigma 3 gas chromatograph equipped with a flame ionization (FID) detector (Perkin-Elmer Co., Norwalk, CT, USA) [15,41]. The sugars determined were fructose, glucose, sucrose, kojibiose, isomaltose and maltose. The last disaccharide includes not only maltose but also nigerose and turanose due to peak overlapping. The fructose/glucose and glucose/water ratios were also calculated. The trisaccharides raffinose, erlose and melezitose were estimated, but due to uncertainty in determination or lack of detection, they were not used in classification algorithms. Analyses were conducted in triplicate.

### 2.8. Classification Using Statistical Multivariate and Machine Learning Algorithms

Once the dataset with the values for all the inputs was obtained, several algorithms were applied to compare their performance to achieve the classification of the samples as accurately as possible. First, in an exploratory analysis, a correlation matrix was obtained. Then, PCA, a well-known unsupervised statistical method, was used to examine the data; it identifies orthogonal directions of maximum variance in the dataset, in decreasing order, and projects the data in a lower-dimensionality space formed of a subset of the components with the highest variance. The orthogonal directions are principal components, which are linear combinations of the original input variables. It is a method for feature reduction and transforms the original independent variables into new axes. PCA helps to identify patterns in data and express them in such a manner to indicate their similarities and differences [27,38]. The k-means algorithm was also applied to the dataset to find clusters among the samples.

The first supervised classification approach was to consider most of the dataset (70%) randomly selected for a training task using 10-fold cross-validation to validate the models and, after finishing training and optimizing the parameters, to utilize the remaining dataset (30%) to test the ability of the best model to accurately classify the samples into their a priori labeled parent classes. Statistical multivariate algorithms applied to this goal were the following: penalized discriminant analysis (PDA) [44], which is a penalized version of Fisher linear discriminant analysis (LDA); the K-NN algorithm, which assumes the similarity between the new sample and available samples within a K distance and places a new sample into the class that is most similar among the available classes. It is non-parametric, i.e., it does not make any assumption on the underlying data. In fact, the used algorithm was a weighted version of K-NN included in the KKNN method of “caret” [45,46]. Other tested classifiers have been high-dimensional discriminant analysis (HDDA) [47], which is a model-based discriminant analysis method that assumes that each class of the dataset resides in a proper Gaussian subspace that is much smaller than the original one, and the function calculates the parameters of each subspace to predict the class of new observation of this kind; nearest shrunken centroids (NSC) [48,49], also known as prediction for microarrays (PAM); C5.0 tree, which is a popular implementation of decision trees; partial least squares (PLS), a multivariate linear regression method that can deal with a large number of predictors, a small sample size and high collinearity among predictors and acts by forming linear combinations of the predictors in a supervised manner; extremely randomized trees (ET) [50]; and shrinkage discriminant analysis (SDA) [51], an algorithm that determines a ranking of predictors by computing CAT scores (correlation-adjusted t-scores) between the group centroids and the pooled mean. Variables were preprocessed and scaled before treatment. Several metrics were used for tuning the algorithm parameters during training/validation. They were logistic loss (log loss), also known as cross-entropy loss, which is a classification loss function that tends to a minimum (the lower limit is zero but there is not a high limit) as model performance increases, meaning that the objective is to minimize the expected loss or risk; accuracy, which increases (the upper limit is 1) as the performance of the classifier increases, that is, when labeled samples are included into their a priori known classes; area under the curve (AUC), which computes the area under the receiver operator characteristic (ROC) curve and also increases (value range 0‒1) with classifier performance; Cohen’s kappa; sensitivity; specificity; and precision, among others. However, log loss was primarily used to measure the performance to build the best model across training/cross-validation, except when this function is not implemented.

Other ML algorithms included in the comparison were ANN (neuralnet library) [52], SVM with linear kernels (SVM library), which is a well-known classifier [53,54], RF (randomForest library) [55], XGBoost (XGBTree library) [56] and extremely randomized trees (ET) [50]. The software used was R and the “classification and regression training” (caret) package [46]. The confusion matrices express the number of samples accurately classified into their parent class or otherwise. To conduct a fair comparison of the different algorithms, the dataset partitions into training and test sets were identical.

## 3. Results

### 3.1. Honey Dataset

For the selected honey samples after microscopy analysis and sensory assessment, a dataset was built using the mean values of each determination. The dataset is summarized in Figures S1 and S2. After microscopic analysis, pollen count was related to nectariferous plants. The box plots of percentages of pollen from the taxa that give the names to the studied unifloral honeys are shown in Figure S2g. Selected samples were as follows: Rosemary honeys that had 20–77% pollen from *R. officinalis* were considered acceptable as it is known as an under-represented pollen. Orange blossom or citrus honeys had a percentage of *Citrus* spp. pollen in the range 10–46%, except in one sample (80%). Citrus honeys are considered unifloral if the pollen of *Citrus* spp. is >10% because it is considered as under-represented. Lavender honeys sowed a percentage of *Lavandula latifolia* or *L. spica* in the range 15–68%. Pollen from *L. stoechas* was usually absent. Additionally, in this honey class, the pollen is considered under-represented. Sunflower honeys had pollen of *H. annuus* in the range 31‒82%. Eucalyptus honeys contained 82‒98% pollen of *Eucalyptus* spp. High counts in this case are usual because *Eucalyptus* pollen is over-represented. Heather honeys encompassed pollen from *Erica* spp. in the range 48–80% (Figure S2g). For forest honey, which is mainly honeydew honey, pollen counts of *Quercus* spp., although always present, are of no interest, as previously commented, because their flowers are non-nectariferous, but they were always examined microscopically for HDE and the presence of pollen from other taxa. HDE presence was scarce. Concerning organoleptic properties, rosemary and orange honeys displayed a light amber color and had a characteristic aroma and taste. Lavender and eucalyptus honeys were light amber but darker than orange or rosemary honeys and had a characteristic aroma and taste. Sunflower honeys had a yellow characteristic and a bright golden-amber color, with a yellow hue and slight tart aroma, and crystalized easily, producing fine crystals. Heather honeys were amber/dark amber with a reddish hue and had a characteristic intense aroma and sour taste and a tendency to crystallize. Forest honeys were also dark amber/dark, had an intense flavor, were slightly bitter and sour and remained liquid even in cool conditions for months.

Figures S1 and S2 show the large variability of the data. Some rosemary and citrus honeys had a high moisture percentage, and the lower water levels were found in eucalyptus and forest honeys, while the remaining honey types exhibited intermediate water contents (Figure S1a). All lavender honeys were well below the 15% limit for sucrose established in the EU Council directive [4]. Forest and heather honeys showed the highest values of electrical conductivity, followed by eucalyptus and lavender/sunflower honeys, while rosemary and citrus displayed the lowest values for this parameter (Figure S1). The pH was also higher in forest and heather honeys than in the remaining honeys. The highest contents of fructose and glucose and the highest glucose/water ratio were found in sunflower honeys, which also had the lowest fructose/glucose ratio; forest honeys showed the lowest levels of both fructose and glucose. On the contrary, maltose, isomaltose and kojibiose contents and the fructose/glucose ratio reached the highest values in forest honeys (Figures S1 and S2). Concerning the color parameters, the largest x values were observed in heather honeys followed by forest honeys, and the minimum x values were observed in rosemary and citrus honeys. However, heather honeys had the lowest mean value for the y and L chromatic coordinates. The largest mean y value was exhibited by sunflowers honeys, and the largest mean L value was observed in citrus honeys (Figure S2).

### 3.2. Statistical and ML Algorithms

**Figure 1:**
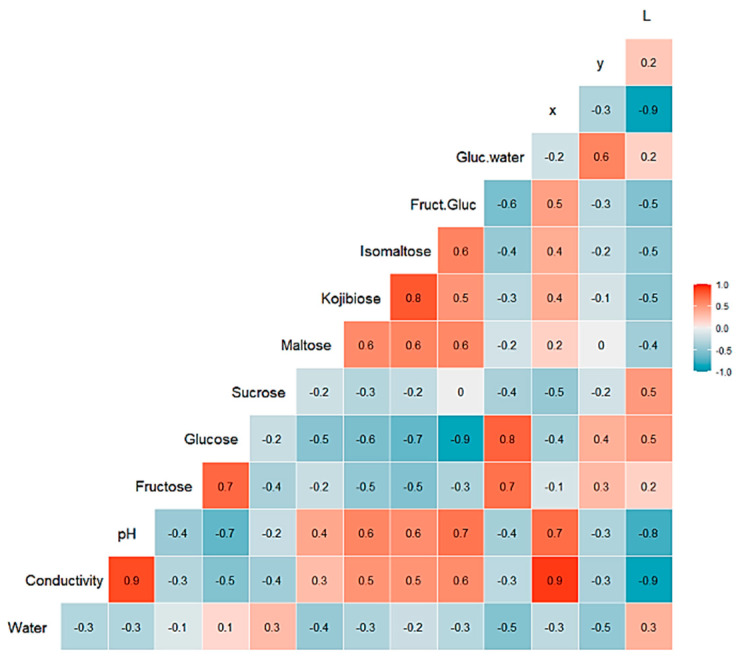
Correlation chart among the 14 predictor variables for the whole dataset. The number in each square is rounded to one figure. The color scale at the right indicates color meaning. Red color means positive correlation; blue color means negative correlation.

**Figure 2:**
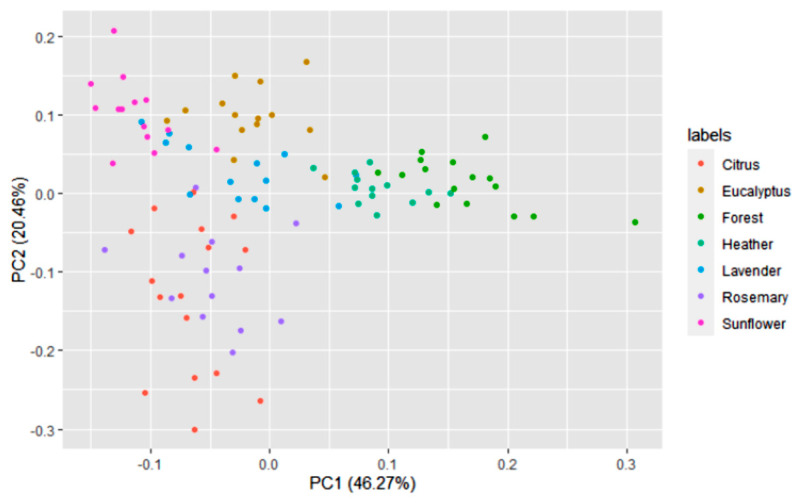
Principal component score plot based on the 14 variables of 100 honey samples according to the botanical origins.

A multivariate statistical study of the dataset was carried out initially. Variables were centered and scaled before statistical treatments. Correlations, PCA and data clustering were performed. A diagram including correlations between all the variables is shown in Figure 1. A score plot of the two principal components can be observed in Figure 2. PC1 and PC2 account for 45.27% and 20.46% of the variance, respectively (overall 66.73%). Heather and forest honeys spread along the positive side of PC1. Sunflower honeys extend on the negative side of PC1, but on the positive side of PC2. All citrus and most rosemary honeys are on the negative side of both PC1 and PC2. Eucalyptus honey samples spread on the positive side of PC2, and most of them are on the negative side of PC1, while most lavender honeys fall on the negative side of PC1, but they spread on both the positive and negative sides of PC2.

**Figure 3:**
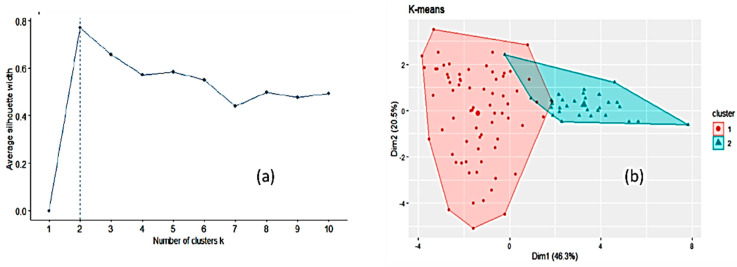
Optimal number of clusters by k-means using the average silhouette width (**a**) and clustering of honey samples by k-means algorithm in two clusters where the two largest symbols are the centroids of each cluster (**b**).

Another unsupervised way to explore the dataset, k-means clustering [57,58], was run to partition the data into a number of clusters using the library “factoextra” in R. All the input variables were taken into account. Two clusters of sizes 70 and 30 were obtained on the basis of the maximum average silhouette width (Figure 3). However, this number of clusters is an estimate and does not mean that only two clusters may exist. The mean values for the variables in each of these two clusters (corresponding to the centroids) are listed in Table S1. Cluster 2 is smaller in size than cluster 1 and is higher than cluster 1 in mean values of electrical conductivity, pH, disaccharides (except sucrose), fructose/glucose ratio and the x chromatic coordinate. When comparing Figure 2 and Figure 3b, cluster 2 seems to encompass forest and heather honeys and cluster 1 the remaining honeys. Forcing the k-means clustering to display seven groups on a two-dimensional plot leads to highly overlapped clusters (Figure S3). The relative importance of the variables was tested using an RF model as a reference (Figure S4). The most important variables are electrical conductivity, the chromatic coordinates, water content, fructose and glucose. The less important variables are glucose/water and fructose/glucose ratios. The Boruta package [59] was applied, and it considered that no variables had to be removed regardless of their relative importance.

Using the approach of supervised modeling, different classifier algorithms were applied to the dataset, which was divided into a training set (70%) and a test set (30%). Ten-fold cross-validation was applied during training with four repetitions. Various metrics (log loss, accuracy, AUC, kappa, sensitivity, specificity, precision, etc.) can be used during training to tune the key parameters of the algorithms in order to find the best ones. The absolute values of metrics vary when training is repeated. Among them, the log loss metric was usually chosen to select the optimal model using the smallest value. For KKNN or KNN (as weights were not relevant), the final value of the tuning parameters used for the optimized model was kmax (maximum number of neighbors) = 5 (Table 1).

For the PDA algorithm, the optimal lambda value was 0.1. For HDDA, the best model had a threshold of 0.300, but this algorithm is not robust and other repetitions led to a different configuration; for SDA, the lowest log loss was obtained with lambda = 0.05, and for PAM, the best model had a threshold = 0.70615. This value can change slightly if the whole treatment is repeated. With PLS, log loss was also used to select the optimal model using the smallest value, and the final value selected for the model was as follows: number of components (ncomp) = 3 (Table 1).

**Figure 4:**
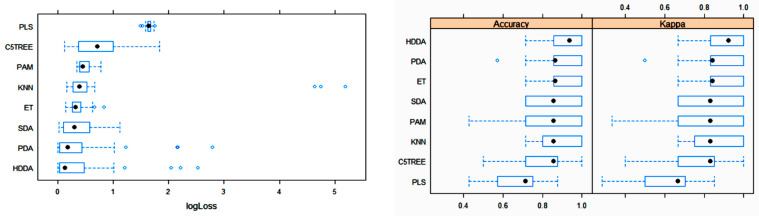
Box plots of log loss, accuracy and kappa values for various machine learning (ML) algorithms after training with 10-fold cross-validation to obtain the best model using the training dataset. Black circles symbolize mean values. PLS: partial least squares; C5TREE: C5.0 tree; PAM: nearest shrunken centroids; KNN: weighted k-nearest neighbors; ET: extremely randomized trees; SDA: shrinkage discriminant analysis; PDA: penalized discriminant analysis; HDDA: high-dimensional discriminant analysis.

The box plots for the three main metric parameters log loss, accuracy and kappa for eight classifiers can be observed in Figure 4.

**Figure 5:**
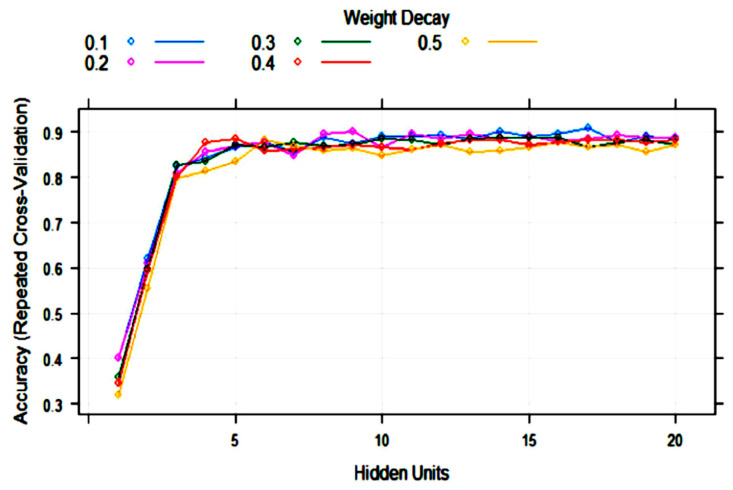
Change in the artificial neural network (ANN) accuracy during training with 10-fold cross-validation with the number of hidden units (nodes) and weight decay.

ANN (single-layer perceptron) was applied to the training set with 10-fold cross-validation. The training process evaluated from 1 to 20 hidden units (neurons) and weight decays from 0.1 to 0.5. After optimization of tuning parameters to maximize the validation accuracy, the best model had 17 hidden units and weight decay = 0.1 (Figure 5). As it can be observed, the variability of accuracy with more than five hidden units is low, ranging from 0.85 to 0.91. This means that repetitions of the treatments can produce different topologies with very similar accuracy.

**Figure 6:**
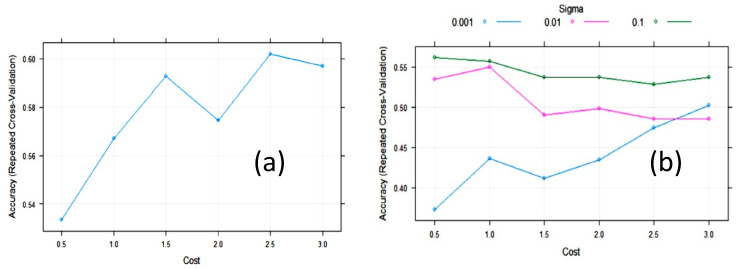
Change in accuracy of (**a**) Support vector machine with linear kernel (SVM_L_) and (**b**) Support vector machine with radial kernel (SVM_R_) during training with 10-fold cross-validation (CV) with the cost function.

The accuracy of the SVM with linear kernels (SVM_L_) algorithm during training with 10-fold cross-validation was maximized, with a value of the cost function of C = 2.5 (Figure 6). The largest value of the accuracy (0.61) was relatively low. In an attempt to improve SVM, we tested SVM with radial basis function kernels (SVM_R_). The final values used for the SVM_R_ model were sigma = 0.1 and C = 0.5 (Figure 6). The accuracy was 0.562, meaning it was not improved. However, the cost value of these algorithms was quite variable on repeated treatments, maintaining the same partition ratio.

**Figure 7:**
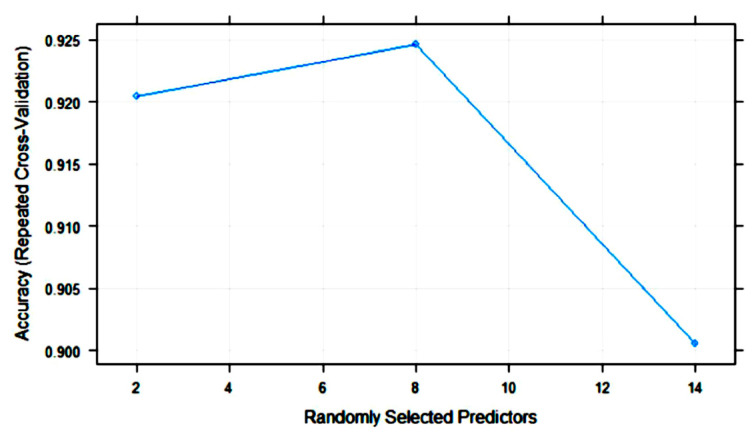
Change in the accuracy of random forest (RF) models with the number of randomly selected predictors (mtry).

Figure 7 shows the variation in RF accuracy throughout training with 10-fold cross-validation. The final values for the RF model were as follows: number of variables randomly sampled as candidates at each split (mtry) = 8; the number of trees (ntry) parameter was 500; the maximum accuracy was 0.9246.

**Figure 8:**
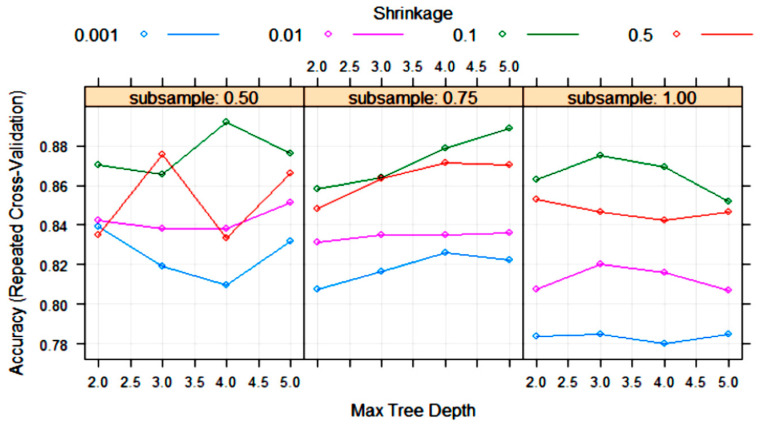
Change in the accuracy of XGBoost algorithm during training with 10-fold cross-validation with the parameters “max tree depth”, “shrinkage (eta)” and “subsample”. Tuning parameters “nrounds”, “gamma”, “colsample_bytree” and “min_child_weight” had constant values of 200, 0, 0.8 and 1, respectively.

The XGBoost tree algorithm has many parameters to tune, although, usually, some of them are held constant. Figure 8 shows the variation in some tuning parameters during the training process. The largest accuracy was obtained with “subsample” = 0.5, “shrinkage (eta)” = 0.1 and “max tree depth” = 4. Other final values for the model were “nrounds” = 200, “gamma” = 0, “colsample_bytree” = 0.8 and “min_child_weight” = 1.

The confusion matrices produced by all the ML models on the test set (30 samples) are listed in Table 2, Table 3 and Table 4. These matrices show the true botanical origin (Reference) in the columns and the predicted classification (Prediction by the models) in the rows. The ideal situation is to have all the samples located on the diagonal cells of the matrix, which would mean that the accuracy is 100%. The overall accuracies obtained with the PDA, SDA, ET, PLS and 5.0 tree algorithms were 90.00%, 86.67%, 86.67%, 73.33% and 76.67%, respectively (Table 2). The overall accuracies obtained with the KKNN, PAM, HDDA, ANN and RF algorithms were 83.33%, 83.33%, 83.33%, 86.67% and 80.00%, respectively (Table 3). The overall accuracies obtained with SVM with linear kernels (SVM_L_), SVM with radial kernels (SVM_R_) and XGBoost were 66.33%, 60.00% and 90.00%, respectively (Table 4).

An accuracy of 100% was not obtained with any model. The largest accuracy was provided by the models obtained with PDA and XGBoost (90%) followed by SDA, ET and ANN. The lowest accuracy was provided by SVM, especially SVM_R_, which failed to correctly classify all heather, lavender and rosemary honeys. All models correctly classified sunflower honeys, and most of them (11) correctly classified all forest honeys. Ten models correctly classified the four heather honeys. Seven models correctly classified all citrus honeys. Only XGBoost classified the four rosemary honeys into their parent groups; no model was able to correctly classify all the lavender honeys. Some lavender honeys were classified as sunflower by XGBoost, SVM, ANN, PAM, RF, HDDA, KKNN, C5.0 tree, PLS and PDA. Other lavender honeys were classified as eucalyptus honeys.

To test the robustness of the overall accuracies, classifications were repeated three more times (the samples included in the training and test sets changed randomly) while maintaining the same splitting ratio (70/30). The box plots of the metrics (log loss, accuracy and kappa) of some optimized models are shown in Figure S5. The results of overall mean accuracies of all the models on the test sets after four repetitions of the whole process (training/10-fold cross-validation), including the ones shown above (Table 2, Table 3 and Table 4), are summarized in Table 5.

As deduced from the results in Table 5, the PDA algorithm had the largest mean overall accuracy on the test set (86.67%), followed by ANN (85.84%), ET, RF and XGBoost (84.17%). The worst performance was rendered by SVM_L_ and SVM_R_ (≤60%). The most stable algorithm was C5.0 tree.

In the case that all samples are used for training with 10-fold cross-validation without separation of a test set, the results are much better with all the models. The training was performed similar to the case of splitting, using the same parameters (log loss, accuracy, kappa) for obtaining the best models (Figure S6). This approach is sometimes found in the literature concerning honey classification, but overfitting is usually a problem. The overall accuracies in this case, according to the confusion matrices (Table S2), were 100% for ET, RF and XGBoost, 97% for PDA and ANN, 95% for C5.0 tree, 92% for SDA, 91% for PAM, 90% for KKNN, 87% for HDDA, 69% for PLS, 66% for SVM_R_ and 56% for SVM_L_.

## 4. Discussion

A variety of factors can influence the variability of the data observed in the dataset. For example, early harvest to increase the amount of honey especially of citrus or rosemary may lead to unripe, very clear products that do not meet legal requirements, although they can be unifloral. Beekeepers or traders can store these crops or blend early with late crops to obtain acceptable products. Early harvest can also affect the sugar content because unripe citrus honey may have more than 20% sucrose. In this case, they cannot be placed directly on the market, although they may be blended with more ripened honeys of the same class to comply with regulations. Late harvest may affect these variables as a large amount of pollen and more variability in the pollen spectrum are expected to occur because bees will go on gathering all available flowers or honeydew and pollen. Therefore, it may be very difficult to obtain unifloral honeys. The more time honey remains inside the combs, the riper the honey is expected to be, with a very low amount of sucrose; the contents of fructose and glucose may vary at low levels or increase, except in the case of honeydew honey, and a safe level of moisture will be reached. Thus, a good balance between early and late harvest should be taken into account by beekeepers to obtain unifloral honeys with the best quality.

In the present study, different ML algorithms using R and the caret package were applied to the same dataset of honeys belonging to seven classes from a single botanical origin collected in Spain. The initial dataset was a matrix of 100 honey samples (rosemary 13, citrus 16, lavender 14, eucalyptus 14, heather 13, sunflower 14 and forest 16) and 14 physiochemical features (water content, electrical conductivity, pH, sugars and colorimetric coordinates). It was partitioned into a training set and a test set.

The first approach to analyze the dataset considered an unsupervised approach. The data were analyzed by PCA and k-means, and two broad clusters with 70 and 30 samples were shown using k-means clustering. The two clusters are too broad to meet the actual honey classes. All variables were used because all were considered important by Boruta. Then, supervised ML approaches were tested. ML classifier algorithms applied were KKNN, PDA, PLS, PAM, HDDA, SDA, C5.0tree, ET, ANN, SVM_L_, SVM_R_, RF and XGBoost.

The metric used for optimizing most models was log loss. Other metric parameters such as accuracy or kappa were also calculated and used instead of log loss for ANN, RF or XGBoost. After training using the same randomly selected dataset (70 samples) and finding the optimal configuration using 10-fold cross-validation, the performance of all models to accurately classify the test samples into their parent classes was compared. All treatments were repeated four times under the same conditions although the samples were randomly distributed in both sets. The performance was not constant among repetitions, and the mean accuracy was considered. The best results were provided by the PDA classifier, which classified the unifloral honeys in the test set within their parent types with 86.67% overall accuracy on average. Good results were also obtained with ANN, ET, HDDA, RF and XGBoost, while SVML and SVMR proved to be the worst. The honeys that have the best chance to be correctly classified are sunflower, forest, heather, eucalyptus and citrus. The correct classification of rosemary honey was hard to carry out, but the most difficult to be appropriately classified were lavender honeys. A low number of samples in the test set can be a problem in making good predictions, especially with samples that have a low percentage of pollen of the putative taxa or have pollen from other nectariferous plants. This happens with lavender, rosemary and citrus honeys, with under-represented pollen [13], and is a problem because they are very appreciated by consumers [60]. It has been reported that pollen analysis can be of limited usefulness for labeling lavender honeys, and analysis of volatiles should be considered as a complementary technique in the case that samples show the characteristic organoleptic properties [21,61].

Application of supervised ML algorithms to the classification of unifloral honeys by botanical origin is an issue of interest. This classification is based on the labeling of the studied samples into classes based on melissopalynology and organoleptic properties. However, microscopic analysis is time-consuming, requires highly specialized personnel and is unable to detect seasonal variation in pollen amounts or fraudulent pollen.

Former attempts at classification were conducted using multivariate statistical discriminant techniques applied to physicochemical features [11], with rather good results. Anjos et al. [36] investigated different ANN configurations to classify the botanical origin of 49 honey samples. Measurements of moisture, electrical conductivity, water activity, ash content, pH, free acidity, colorimetric coordinates and total phenol content were used as input variables. It was concluded that the botanical origin of honey can be reliably and quickly known from the colorimetric information and the electrical conductivity of the honey, which agrees with our results. Another report [24] showed the results obtained with a similar set of variables, although including a large phenolic profile, to classify acacia, tilia (linden), sunflower, honeydew and polyfloral honeys of Romanian origin (50 samples) labeled by pollen analysis into their parent classes by using LDA and ANN as classifiers. LDA correctly classified 92.0% of the samples. An ANN with two hidden layers classified 94.8% of the honey samples into their botanical origin. However, all samples from each class were used to reach these accuracy rates. In the present paper, a test set was used to calculate the percentage of correctly assigned origins, and we obtained higher accuracy rates using all the samples. Popek et al. [37] were able to correctly classify nearly all their samples according to their botanical origin using CART. They obtained good results using all 72 samples (9 samples × 8 classes) under treatment (rape, acacia, heather, linden, buckwheat, honeydew, nectar-honeydew and multifloral honeys).

Authentication of honey origin using ML algorithms and nondestructive analytical techniques has been reported. In this way, ATR-FTIR spectra of 130 Serbian samples belonging to acacia, linden and sunflower honeys were treated by SVM, and the predictability rate was high [27], although the classes only totaled three, and the method of carrying out sample labeling was omitted. In our treatment, SVM was not useful. Ciulu et al. [62] reported on the usage of ATR-FTIR spectra and processing by RF to this aim. Eighty samples belonging to four different floral origins were considered: strawberry tree, asphodel, thistle and eucalyptus. Training an RF on the IR spectra allowed achieving an average accuracy of 87% in a cross-validation setting. This is approximately the same accuracy rate obtained in our study using different variables. FT-Raman spectra combined with PCA or LDA have also proved to be useful to classify monofloral honeys with a high degree of accuracy [29,30,31].

Using NIR (850–2500 nm), classification of 119 Italian honey samples encompassing acacia, linden and chestnut unifloral honeys and multifloral honeys was attempted by PLS and SVM with linear kernels using cross-validation of the NIR spectra [63]. Pollen analysis was not used for labeling. SVM provided better classification scores than PLS contrary to what happens in our case. An additional approach was to apply Boruta for feature selection, but the accuracy was not improved. Splitting of the dataset into a training/CV set and an independent test set was not carried out, meaning that confusion matrices included all samples, which obviously improves the success rate. Linden honeys failed to be correctly classified, which might be due to the low number of samples of that class. NIR was also the source of input variables to classify five types of Chinese unifloral honeys by application of Mahalanobis distance discriminant analysis (MD-DA) and a backpropagation artificial neural network (BP-ANN) [64]. By the MD-DA model, overall correct classification rates were 87.4% and 85.3% for the calibration and validation samples, respectively, while the ANN model resulted in having total correct classification rates of 90.9% and 89.3% for the calibration and validation sets, respectively. Pollen analysis was not employed for origin assignation to honeys. Minaei et al. [28] used VIS–NIR hyperspectral images of 52 samples of five classes of unifloral honeys and, after a reduction in dimensionality, applied RBF networks (a type of ANN with several distinctive features), RF and SVM for classification. The test set had 20 samples, and the remaining 32 samples were used for training. The first ML rendered 92% accuracy, while SVM and RF returned accuracies of 84 and 89%, respectively. A problem related to this technique is the variability in color with time.

Other types of input variables within the group of nondestructive methodologies are based on sensors able to mimic organoleptic perceptions such as the electronic nose (E-nose) [34,65] and the electronic tongue [35]. The E-nose generates signals corresponding to volatile and semivolatile compounds from honeys that, after being processed by ML algorithms, have the ability to carry out correct classifications. Benedetti et al. [65] studied 70 samples ascribed to three unifloral origins, which were certificated by pollen analysis. First, a PCA of samples indicated the main components, and then an ANN was generated that, after optimization by cross-validation, was able to accurately classify all samples of the test set. An electronic tongue was reported to correctly classify acacia, chestnut and honeydew honeys after application of an ANN to signals from the device [35].

Thus, the application of ML algorithms to classification of unifloral honeys has been increasing in recent years, and it is expected that it will go on increasing in the future. However, a systematic comparison of the main ML algorithms to reach this goal as it is presented in this study has not been reported to date. The PDA algorithm was the best, but others such as ANN, SDA, RF, ET or HDDA can also be useful to perform accurate classifications based on the variables from the dataset. SVM worked badly with all repetitions on the datasets. Failure in obtaining larger accuracy rates is due to some honey classes such as lavender, rosemary or citrus with under-represented pollen grains. Good marker parameters should be found and used to improve the classification of these honeys that have not been included in most studies using ML algorithms for prediction. To our knowledge, this is the first time that most of the compared algorithms in the present study (for example, PDA, HDDA, SDA, C5.0, ET, XGBoost) have been used for the goal of classification of unifloral honeys. It is expected that the comparison of the performance of the ML algorithms applied here may be useful not only for research on the topic of honey classification by origin but also for research on other kinds of foods.

## 5. Conclusions

A comparison of 13 ML algorithms on a dataset of one hundred honeys harvested in Spain and belonging to seven unifloral classes was performed using 14 physicochemical parameters. The ML algorithms were built by splitting the dataset into a training set (70%) and a test set (30%) and optimizing the configuration by 10-fold cross-validation using several parameters, but mainly log loss. The optimized models were tested on the test set to record the overall and partial accuracies in the right classification of samples into their parent classes. The whole process was repeated three times, and the results were averaged. The best accuracies were provided by the PDA algorithm, (86.67%), followed by ANN (85.56%), SDA and RF (83.33%). The worst results were rendered by SVM with radial and linear kernels (53–60%). Most algorithms correctly classified forest, sunflower and heather honeys. Orange blossom and eucalyptus honey samples were partly misclassified by some models; rosemary honeys were partly misclassified by all models, except XGBoost, while lavender honeys were the most difficult to be included into their parent groups. Most the algorithms studied here have not been applied previously to the issue of honey classification, and they can likely be useful for such a task in future research such as the inclusion of more unifloral honey types and a multifloral honey class. Moreover, other parameters (among them those obtained by FT-IR, FT-Raman or NIR spectroscopy nondestructive techniques) can be included in the datasets and tested to improve the accuracy of the classification task as much as possible.

## Figures and Tables

**Table 1:** Model optimization using the classifier algorithms. Log loss values are means of 10-fold cross-validation.

Algorithm	Tuning Parameter	Mean Log Loss Values
KKNN	Kmax = 5	0.8339319
	Kmax = 7	0.9017721
	Kmax = 9	0.9808674
PDA	Lambda = 1	0.5689435
	Lambda = 0.0001	0.5687306
	Lambda = 0.1	0.4611719
HDDA	Thershold = 0.05	0.4360396
	Thershold = 0.175	1.3732500
	Thershold = 0.300	1.0080708
SDA	Lambda = 0.0	0.6320813
	Lambda = 0.5	0.3968958
	Lambda = 1.0	0.4908678
PAM	Threshold = 0.7608929	0.4986565
	Threshold = 11.0329476	1.9483062
	Threshold = 21.3050022	1.9483062
PLS	Ncomp = 1	1.826913
	Ncomp = 2	1.733439
	Ncomp = 3	1.643669
C5.0 tree		0.7482527
ET		0.3590714

KKNN: weighted k-nearest neighbors; PDA: penalized discriminant analysis; HDDA: high-dimensional discriminant analysis; SDA: shrinkage discriminant analysis; PAM: nearest shrunken centroids; PLS: partial least squares; ET: extremely randomized trees.

**Table 2:** Confusion matrices of various classifier algorithms (PDA, SDA, ET, PLS, C5.0 tree) on the test set. The number of honey samples in this set was as follows: citrus (5), eucalyptus (4), forest (5), heather (4), lavender (4), rosemary (4) and sunflower (4).

		Reference						
	Prediction	Citrus	Eucalyptus	Forest	Heather	Lavender	Rosemary	Sunflower
PDA	Citrus	5	0	0	0	0	1	0
	Eucalyptus	0	4	0	0	1	0	0
	Forest	0	0	5	0	0	0	0
	Heather	0	0	0	4	0	0	0
	Lavender	0	0	0	0	2	0	0
	Rosemary	0	0	0	0	0	3	0
	Sunflower	0	0	0	0	1	0	4
SDA	Citrus	4	0	0	0	0	1	0
	Eucalyptus	0	4	0	0	1	0	0
	Forest	0	0	5	0	0	0	0
	Heather	0	0	0	4	0	0	0
	Lavender	0	0	0	0	3	1	0
	Rosemary	1	0	0	0	0	2	0
	Sunflower	0	0	0	0	0	0	4
ET	Citrus	4	0	0	0	0	0	0
	Eucalyptus	0	4	0	0	2	0	0
	Forest	0	0	5	0	0	0	0
	Heather	0	0	0	4	0	0	0
	Lavender	0	0	0	0	2	1	0
	Rosemary	1	0	0	0	0	3	0
	Sunflower	0	0	0	0	0	0	4
PLS	Citrus	5	0	0	0	0	3	0
	Eucalyptus	0	3	0	0	0	0	0
	Forest	0	0	5	0	0	1	0
	Heather	0	0	0	4	0	0	0
	Lavender	0	0	0	0	1	0	0
	Rosemary	0	0	0	0	0	0	0
	Sunflower	0	1	0	0	3	0	4
C5.0 tree	Citrus	4	1	0	0	0	1	0
	Eucalyptus	0	2	0	0	2	0	0
	Forest	0	0	5	0	0	0	0
	Heather	0	0	0	4	0	0	0
	Lavender	0	1	0	0	1	0	0
	Rosemary	0	0	0	0	0	3	0
	Sunflower	1	0	0	0	1	0	4

**Table 3:** Confusion matrices of various classifier algorithms (KKNN, PAM, HDDA, ANN and RF) on the test set. The number of honey samples was the same as that indicated in Table 2.

		Reference						
	Prediction	Citrus	Eucalyptus	Forest	Heather	Lavender	Rosemary	Sunflower
KKNN	Citrus	4	0	0	0	0	1	0
	Eucalyptus	0	4	0	0	0	0	0
	Forest	0	0	5	0	0	0	0
	Heather	0	0	0	4	0	0	0
	Lavender	0	0	0	0	1	0	0
	Rosemary	1	0	0	0	0	3	0
	Sunflower	0	0	0	0	3	0	4
PAM	Citrus	5	0	0	0	0	1	0
	Eucalyptus	0	3	0	0	1	0	0
	Forest	0	0	5	0	0	0	0
	Heather	0	0	0	4	0	0	0
	Lavender	0	0	0	0	2	0	0
	Rosemary	0	0	0	0	0	3	0
	Sunflower	0	1	0	0	1	0	4
HDDA	Citrus	4	0	0	0	0	1	0
	Eucalyptus	0	4	0	0	1	0	0
	Forest	0	0	4	0	0	0	0
	Heather	0	0	1	4	0	0	0
	Lavender	0	0	0	0	2	0	0
	Rosemary	0	0	0	0	0	3	0
	Sunflower	1	0	0	0	1	0	4
ANN	Citrus	5	0	0	0	0	1	0
	Eucalyptus	0	4	0	0	0	0	0
	Forest	0	0	5	0	0	0	0
	Heather	0	0	0	4	0	0	0
	Lavender	0	0	0	0	1	0	0
	Rosemary	0	0	0	0	0	3	0
	Sunflower	0	0	0	0	3	0	4
RF	Citrus	5	1	0	0	0	1	0
	Eucalyptus	0	2	0	0	1	0	0
	Forest	0	0	5	1	0	0	0
	Heather	0	0	0	3	0	0	0
	Lavender	0	1	0	0	2	0	0
	Rosemary	0	0	0	0	0	3	0
	Sunflower	0	0	0	0	1	0	4

**Table 4:** Confusion matrices of various classifier algorithms (SVM_L_, SVN_R_ and XGBoost) on the test set. The number of honey samples was the same as that indicated in Table 2.

		Reference						
	Prediction	Citrus	Eucalyptus	Forest	Heather	Lavender	Rosemary	Sunflower
SVM_L_	Citrus	3	0	0	0	0	1	0
	Eucalyptus	0	4	0	1	1	0	0
	Forest	0	0	5	2	0	0	0
	Heather	0	0	0	0	0	0	0
	Lavender	0	0	0	1	0	0	0
	Rosemary	2	0	0	0	1	3	0
	Sunflower	0	0	0	0	2	0	4
SVM_R_	Citrus	5	0	0	1	0	4	0
	Eucalyptus	0	4	0	0	2	0	0
	Forest	0	0	5	0	0	0	0
	Heather	0	0	0	0	0	0	0
	Lavender	0	0	0	3	0	0	0
	Rosemary	0	0	0	0	0	0	0
	Sunflower	0	0	0	0	2	0	4
XGB	Citrus	5	0	0	0	0	0	0
	Eucalyptus	0	4	0	0	0	0	0
	Forest	0	0	4	0	0	0	0
	Heather	0	0	1	4	0	0	0
	Lavender	0	0	0	0	2	0	0
	Rosemary	0	0	0	0	0	4	0
	Sunflower	0	0	0	0	2	0	4

**Table 5:** Overall accuracy of ML models for classification of honey samples in the test sets.

ML Algorithm	Overall Accuracy per Test				Mean Overall Accuracy
	Test 1	Test 2	Test 3	Test 4	
PLS	0.7333	0.6667	0.6333	0.7000	0.6833
C5.0 tree	0.7667	0.7667	0.7667	0.8000	0.7750
KKNN	0.8333	0.8333	0.7000	0.8000	0.7916
PAM	0.8333	0.8333	0.6667	0.8667	0.8000
PDA	0.9000	0.9333	0.7667	0.8667	0.8667
SDA	0.8667	0.8667	0.7667	0.8333	0.8333
ET	0.8333	0.8667	0.7667	0.9000	0.8417
HDDA	0.8333	0.8667	0.7667	0.9000	0.8417
ANN	0.8667	0.9333	0.7667	0.8667	0.8584
RF	0.8000	0.8333	0.8667	0.8667	0.8417
SVML	0.6333	0.4667	0.5000	0.6667	0.5667
SVMR	0.6000	0.6667	0.5333	0.5667	0.5917
XGBoost	0.9000	0.8333	0.7000	0.9333	0.8417

## Data Availability

The data supporting the results can be found in Supplementary Materials or by petition to the corresponding author.

## Supplementary Materials

The following are available online at https://www.mdpi.com/article/10.3390/foods10071543/s1, Figure S1: Box plots of some variables in the honey dataset: (a) water content (%); (b) electrical conductivity (µs/cm); (c) pH; (d) fructose content (%); (e) glucose content (%); (f) sucrose content (%); (g) maltose content (%); (h) isomaltose content (%), Figure S2: Box plots of some variables in the honey dataset: (a) kojibiose content (%); (b) fructose/glucose ratio; (c) glucose/water ratio; (d) chromatic coordinate x; (e) chromatic coordinate y; (f) chromatic coordinate L; (g) percentage of pollen from the characteristic taxa giving the name to each unifloral honey (this variable is not included in the dataset), Figure S3: Clustering of the honey dataset in 7 clusters by k-means. The largest symbols correspond to the centroids of each cluster, Figure S4: Plot of the importance of the variables from the honey dataset, as estimated by an RF model, Figure S5: Box plots of metric values of several ML algorithms by optimization throughout training with 10-fold cross-validation of the honey dataset with splitting into training and test sets obtained in three additional repetitions: (a), (b) and (c) log loss of repetitions 2, 3 and 4; (d), (e) and (f) accuracy and kappa of repetitions 2, 3 and 4. Black circles symbolize mean values, Figure S6: Box plots of (a) log loss, (b) accuracy and kappa values of several ML algorithms throughout the training with 10-fold cross-validation of the honey dataset without partitioning into training and test sets. Black circles symbolize mean values, Table S1: Mean values for the predictor variables in the two clusters obtained by k-means, Table S2: Confusion matrices obtained with all honeys in the dataset without splitting into training and test sets using 10-fold cross-validation.
