# Supplementary material for: Comparative Study of Several Machine Learning Algorithms for Classification of Unifloral Honeys
Source: Foods. 2021 Jul 3;10(7):1543. doi: 10.3390/foods10071543 (PMC8303996; doi:10.3390/foods10071543)
Supplement: Supplementary file 1 [file foods-10-01543-s001.zip › Figure S1.pdf]

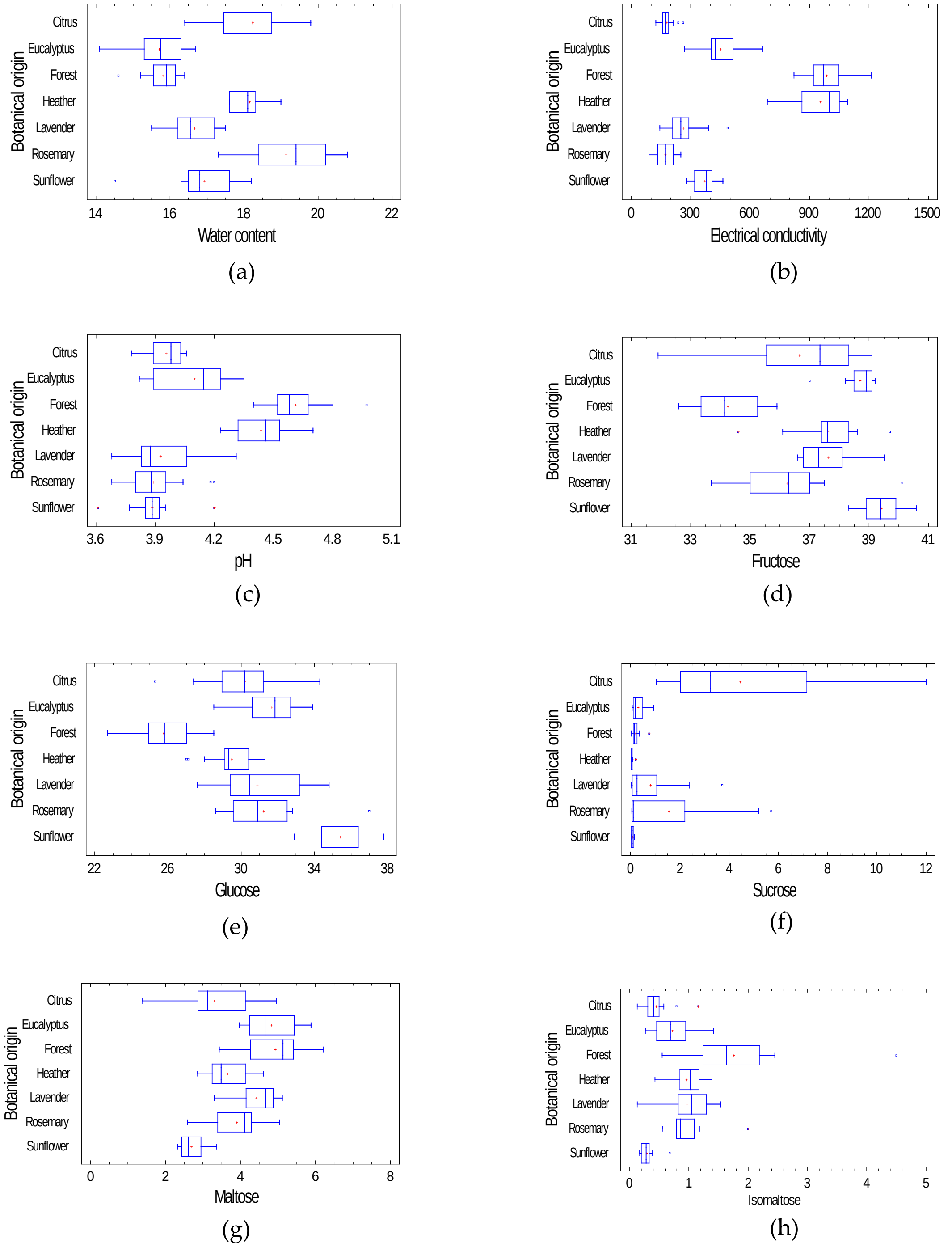

Figure S1. Box plots of some variables in the honey dataset: (a) water content (%); (b) electrical conductivity (μs/cm); (c) pH; (d) fructose content (%); (e) glucose content (%); (f) sucrose content (%); (g) maltose content (%); (h) isomaltose content (%).
