# Supplementary material for: Comparative Study of Several Machine Learning Algorithms for Classification of Unifloral Honeys
Source: Foods. 2021 Jul 3;10(7):1543. doi: 10.3390/foods10071543 (PMC8303996; doi:10.3390/foods10071543)
Supplement: Supplementary file 1 [file foods-10-01543-s001.zip › Figure S2.pdf]

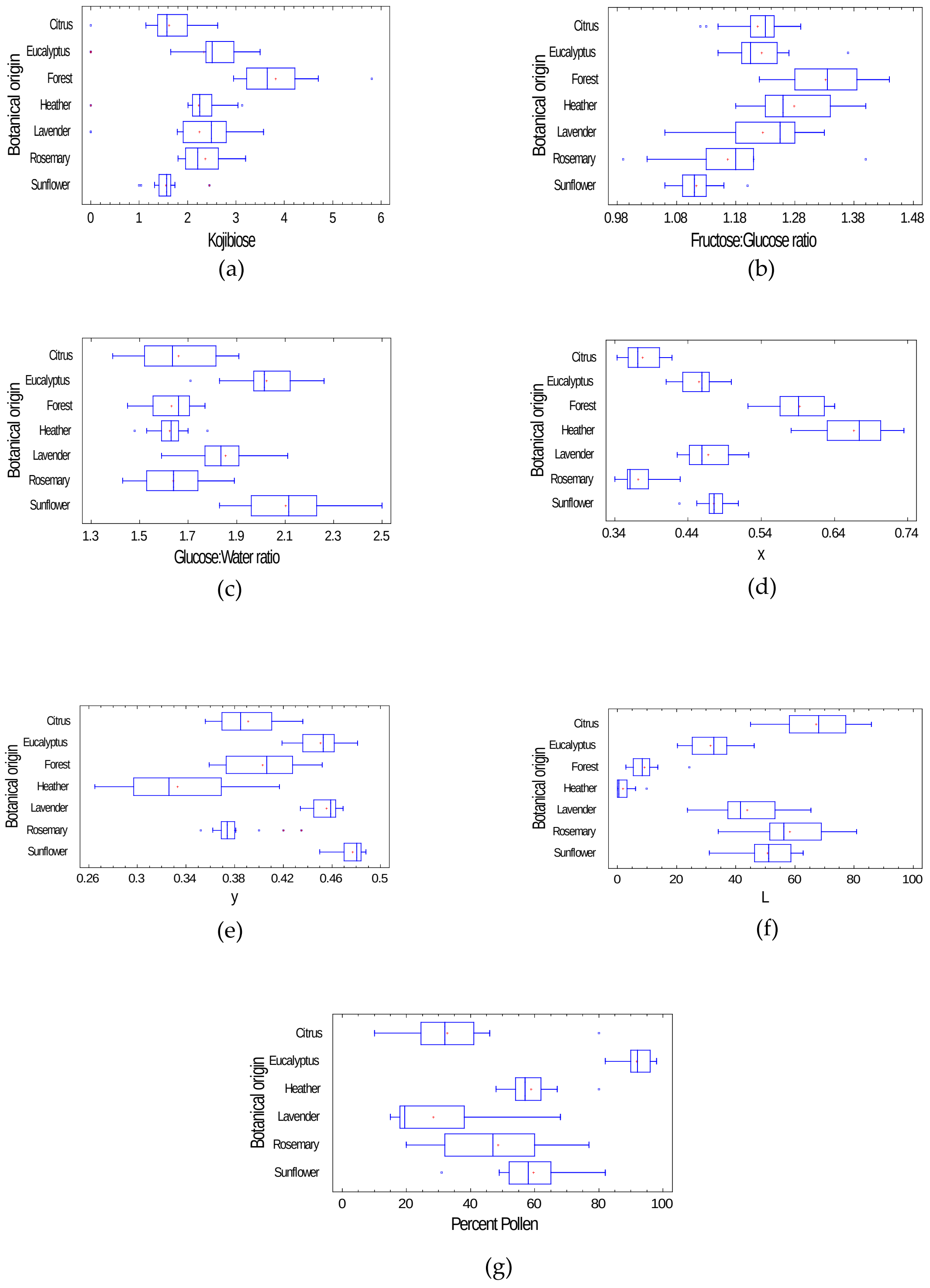

Figure S2. Box plots of some variables in the honey dataset: (a); kojibiose content (%); (b) fructose/glucose ratio; (c) glucose/water ratio; (d) chromatic coordinate x; (e) chromatic coordinate y; (f) chromatic coordinate L; (g) percentage of pollen from the characteristic taxa giving the name to each unifloral honey (this variable is not included in the dataset).
