# Supplementary material for: Comparative Study of Several Machine Learning Algorithms for Classification of Unifloral Honeys
Source: Foods. 2021 Jul 3;10(7):1543. doi: 10.3390/foods10071543 (PMC8303996; doi:10.3390/foods10071543)
Supplement: Supplementary file 1 [file foods-10-01543-s001.zip › Figure S3.pdf]

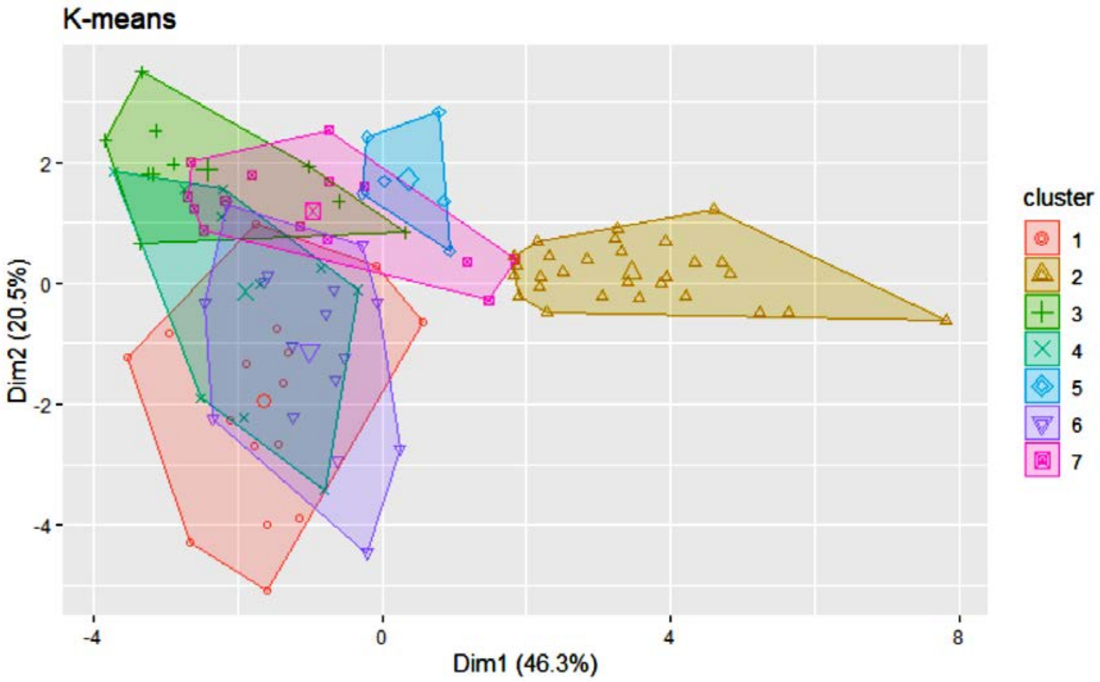


**Figure S3.** Clustering of the honey data set in 7 clusters by k-means. The largest symbols correspond to the centroids of each cluster.
