# Supplementary material for: Comparative Study of Several Machine Learning Algorithms for Classification of Unifloral Honeys
Source: Foods. 2021 Jul 3;10(7):1543. doi: 10.3390/foods10071543 (PMC8303996; doi:10.3390/foods10071543)
Supplement: Supplementary file 1 [file foods-10-01543-s001.zip › Figure S5.pdf]

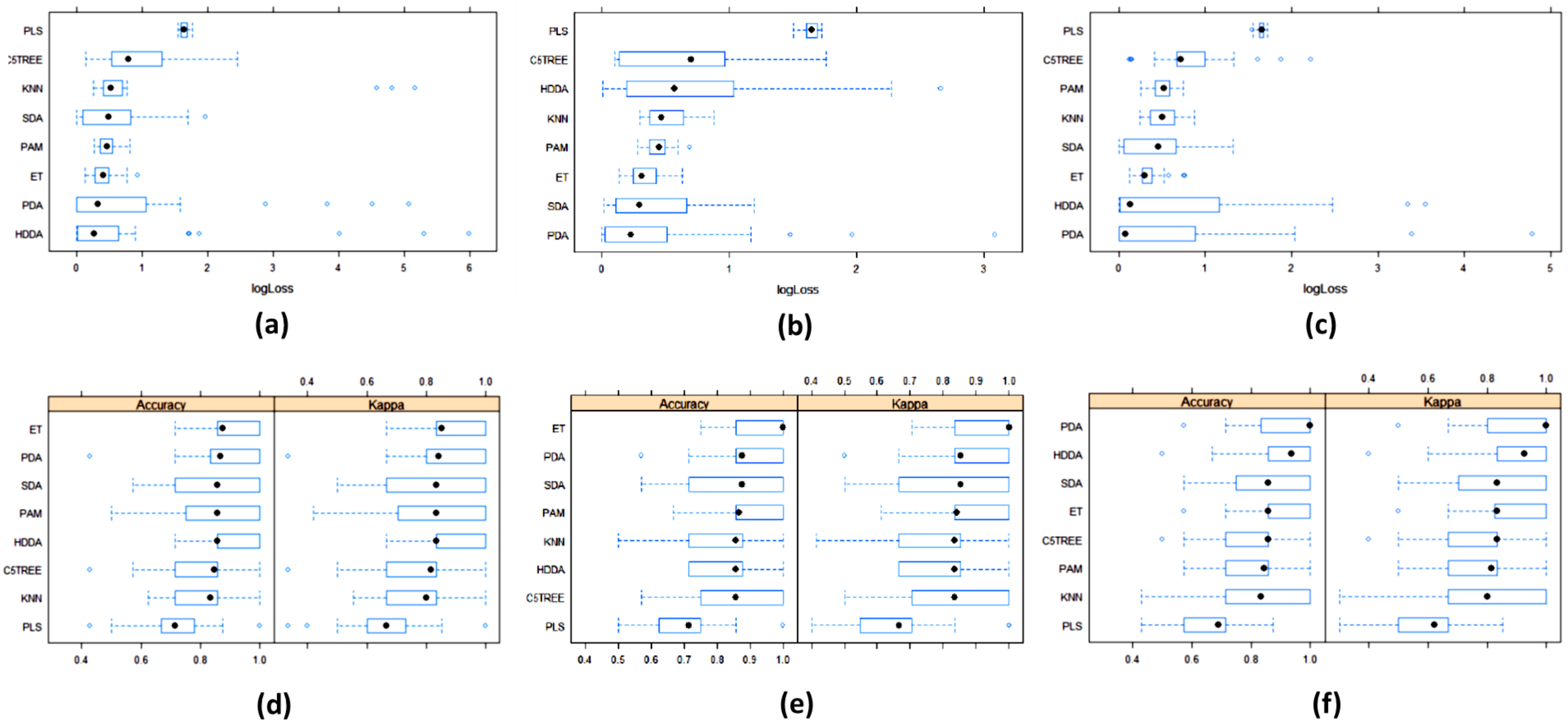

Figure S5. Box plots of metric values of several ML algorithms by optimization throughout training with 10-fold cross-validation of the honey dataset with splitting into training as test sets obtained in three additional repetitions: **(a)**, **(b)** and **(c)** Log loss of repetitions 2, 3 and 4; **(d)**, **(e)** and **(f)** Accuracy and kappa of repetitions 2, 3 and 4. Black circles symbolize mean values.
