# Supplementary material for: Comparative Study of Several Machine Learning Algorithms for Classification of Unifloral Honeys
Source: Foods. 2021 Jul 3;10(7):1543. doi: 10.3390/foods10071543 (PMC8303996; doi:10.3390/foods10071543)
Supplement: Supplementary file 1 [file foods-10-01543-s001.zip › Figure S6.pdf]

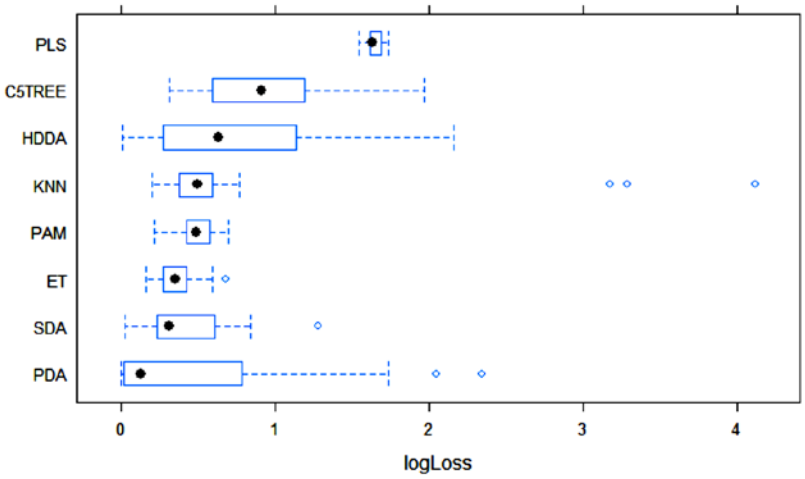

(**a**)

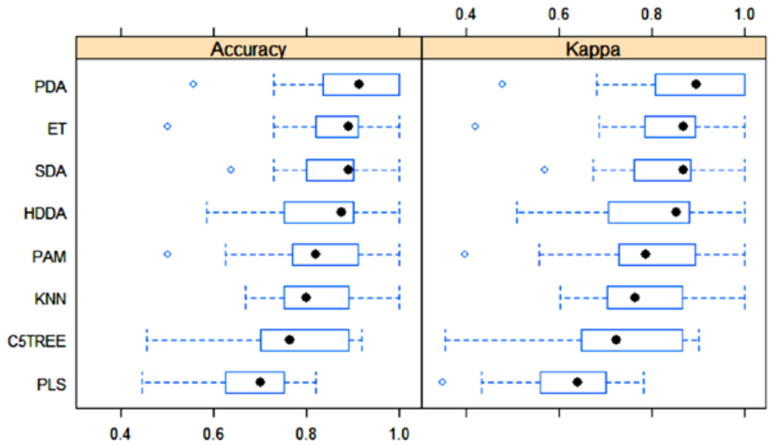

(**b**)

Figure S6. Box plots of (**a**) log loss, (**b**) accuracy and kappa values of several ML algorithms throughout the training with 10-fold cross-validation of the honey dataset without partitioning into training as test sets. Black circles symbolize mean values.
