# Supplementary material for: Comparative Study of Several Machine Learning Algorithms for Classification of Unifloral Honeys
Source: Foods. 2021 Jul 3;10(7):1543. doi: 10.3390/foods10071543 (PMC8303996; doi:10.3390/foods10071543)
Supplement: Supplementary file 1 [file foods-10-01543-s001.zip › Table S1.pdf]

Table S1. Mean values for the predictor variables in the two clusters obtained by k-means.

Variable	Cluster 1	Cluster 2
Water content (%)	17.35571	16.81667
Electrical conductivity (μS/cm)	277.7537	961.8333
pH	3.947429	4.524667
Fructose content (%)	37.69571	35.87333
Glucose content (%)	31.82286	27.59667
Sucrose content (%)	1.541585	0.1422333
Maltose content (%)	3.779000	4.345667
Kojibiose content (%)	1.998429	3.086333
Isomaltose content (%)	0.6707143	1.3756667
F/G ratio	1.190089	1.304986
G/W ratio	1.849119	1.643134
x	0.4270286	0.6124667
y	0.4297714	0.3766333
L	51.23211	6.43750
