# Supplementary material for: Comparative Study of Several Machine Learning Algorithms for Classification of Unifloral Honeys
Source: Foods. 2021 Jul 3;10(7):1543. doi: 10.3390/foods10071543 (PMC8303996; doi:10.3390/foods10071543)
Supplement: Supplementary file 1 [file foods-10-01543-s001.zip › Table S2.pdf]

Table S2. Confusion matrixes obtained with all honeys in the data set without splitting into training and test set using 10-fold cross-validation.

PLS

Prediction \ Reference	Citrus	Eucalyptus	Forest	Heather	Lavender	Rosemary	Sunflower
Citrus	15	0	0	0	1	12	0
Eucalyptus	0	8	0	0	4	0	0
Forest	0	2	16	0	2	0	0
Heather	0	0	0	13	0	0	0
Lavender	0	0	0	0	3	0	0
Rosemary	0	0	0	0	0	0	0
Sunflower	1	4	0	0	4	1	14

C5.0 tree

Prediction \ Reference	Citrus	Eucalyptus	Forest	Heather	Lavender	Rosemary	Sunflower
Citrus	15	0	0	0	0	2	0
Eucalyptus	0	11	0	0	0	0	0
Forest	0	0	16	0	0	0	0
Heather	0	0	0	13	0	0	0
Lavender	0	3	0	0	11	1	0
Rosemary	0	0	0	0	0	10	0
Sunflower	1	0	0	0	3	0	14

PAM

Prediction \ Reference	Citrus	Eucalyptus	Forest	Heather	Lavender	Rosemary	Sunflower
Citrus	14	0	0	0	0	2	0
Eucalyptus	0	13	0	0	0	0	0
Forest	0	0	16	0	0	0	0
Heather	0	0	0	13	0	0	0
Lavender	0	1	0	0	10	0	0
Rosemary	2	0	0	0	0	11	0
Sunflower	0	0	0	0	4	0	14

KKNN

Prediction \ Reference	Citrus	Eucalyptus	Forest	Heather	Lavender	Rosemary	Sunflower
Citrus	15	0	0	0	0	2	0
Eucalyptus	0	11	0	0	0	0	0
Forest	0	0	16	0	0	0	0
Heather	0	0	0	13	0	0	0
Lavender	0	3	0	0	11	1	0
Rosemary	0	0	0	0	0	10	0
Sunflower	1	0	0	0	3	0	14

Table S2. Continued

PDA

Prediction \ Reference	Citrus	Eucalyptus	Forest	Heather	Lavender	Rosemary	Sunflower
Citrus	16	0	0	0	0	1	0
Eucalyptus	0	14	0	0	0	0	0
Forest	0	0	16	1	0	0	0
Heather	0	0	0	12	0	0	0
Lavender	0	0	0	0	13	0	0
Rosemary	0	0	0	0	0	12	0
Sunflower	0	0	0	0	1	0	14

SDA

Prediction \ Reference	Citrus	Eucalyptus	Forest	Heather	Lavender	Rosemary	Sunflower
Citrus	13	0	0	0	0	2	0
Eucalyptus	0	14	0	0	0	0	0
Forest	0	0	16	1	0	0	0
Heather	0	0	0	12	0	0	0
Lavender	1	0	0	0	13	1	0
Rosemary	2	0	0	0	0	10	0
Sunflower	0	0	0	0	1	0	14

ET

Prediction \ Reference	Citrus	Eucalyptus	Forest	Heather	Lavender	Rosemary	Sunflower
Citrus	16	0	0	0	0	0	0
Eucalyptus	0	14	0	0	0	0	0
Forest	0	0	16	0	0	0	0
Heather	0	0	0	13	0	0	0
Lavender	0	0	0	0	14	0	0
Rosemary	0	0	0	0	0	13	0
Sunflower	0	0	0	0	0	0	14

HDDA

Prediction \ Reference	Citrus	Eucalyptus	Forest	Heather	Lavender	Rosemary	Sunflower
Citrus	13	0	0	0	0	2	0
Eucalyptus	0	11	0	0	2	0	0
Forest	0	0	16	1	0	0	0
Heather	0	0	0	12	0	0	0
Lavender	1	3	0	0	11	1	0
Rosemary	2	0	0	0	0	10	0
Sunflower	0	0	0	0	1	0	14

Table S2. Continued

ANN

Prediction \ Reference	Citrus	Eucalyptus	Forest	Heather	Lavender	Rosemary	Sunflower
Citrus	15	0	0	0	0	1	0
Eucalyptus	0	14	0	0	0	0	0
Forest	0	0	16	0	0	0	0
Heather	0	0	0	13	0	0	0
Lavender	0	0	0	0	13	0	0
Rosemary	1	0	0	0	0	12	0
Sunflower	0	0	0	0	1	0	14

RF

Prediction \ Reference	Citrus	Eucalyptus	Forest	Heather	Lavender	Rosemary	Sunflower
Citrus	16	0	0	0	0	0	0
Eucalyptus	0	14	0	0	0	0	0
Forest	0	0	16	0	0	0	0
Heather	0	0	0	13	0	0	0
Lavender	0	0	0	0	14	0	0
Rosemary	0	0	0	0	0	13	0
Sunflower	0	0	0	0	0	0	14

SVM_L

Prediction \ Reference	Citrus	Eucalyptus	Forest	Heather	Lavender	Rosemary	Sunflower
Citrus	11	0	0	0	0	3	0
Eucalyptus	0	14	0	1	6	0	1
Forest	0	0	16	7	0	0	0
Heather	0	0	0	3	0	0	0
Lavender	0	0	0	2	0	0	0
Rosemary	5	0	0	0	8	10	11
Sunflower	0	0	0	0	0	0	2

SVM_R

Prediction \ Reference	Citrus	Eucalyptus	Forest	Heather	Lavender	Rosemary	Sunflower
Citrus	8	0	0	0	0	2	0
Eucalyptus	0	13	0	0	5	1	1
Forest	0	0	16	0	1	0	0
Heather	0	0	0	10	0	0	0
Lavender	0	0	0	3	0	0	0
Rosemary	8	1	0	0	7	10	4
Sunflower	0	0	0	0	1	0	9

XGBoost

Prediction	Reference Citrus	Eucalyptus	Forest	Heather	Lavender	Rosemary	Sunflower
Citrus	16	0	0	0	0	0	0
Eucalyptus	0	14	0	0	0	0	0
Forest	0	0	16	0	0	0	0
Heather	0	0	0	13	0	0	0
Lavender	0	0	0	0	14	0	0
Rosemary	0	0	0	0	0	13	0
Sunflower	0	0	0	0	0	0	14
